# TasselLFANet: a novel lightweight multi-branch feature aggregation neural network for high-throughput image-based maize tassels detection and counting

**DOI:** 10.3389/fpls.2023.1158940

**Published:** 2023-04-14

**Authors:** Zhenghong Yu, Jianxiong Ye, Cuina Li, Huabing Zhou, Xun Li

**Affiliations:** ^1^ College of Robotics, Guangdong Polytechnic of Science and Technology, Zhuhai, Guangdong, China; ^2^ Meteorological Observation Centre, China Meteorological Administration, Beijing, China; ^3^ Department of Computer Science and Engineering, Wuhan Institute of Technology, Wuhan, China

**Keywords:** maize tassels, detection and counting, lightweight, feature aggregation, neural network

## Abstract

Accurately and rapidly counting the number of maize tassels is critical for maize breeding, management, and monitoring the growth stage of maize plants. With the advent of high-throughput phenotyping platforms and the availability of large-scale datasets, there is a pressing need to automate this task for genotype and phenotype analysis. Computer vision technology has been increasingly applied in plant science, offering a promising solution for automated monitoring of a large number of plants. However, the current state-of-the-art image algorithms are hindered by hardware limitations, which compromise the balance between algorithmic capacity, running speed, and overall performance, making it difficult to apply them in real-time sensing field environments. Thus, we propose a novel lightweight neural network, named TasselLFANet, with an efficient and powerful structure for accurately and efficiently detecting and counting maize tassels in high spatiotemporal image sequences. Our proposed approach improves the feature-learning ability of TasselLFANet by adopting a cross-stage fusion strategy that balances the variability of different layers. Additionally, TasselLFANet utilizes multiple receptive fields to capture diverse feature representations, and incorporates an innovative visual channel attention module to detect and capture features more flexibly and precisely. We conducted a series of comparative experiments on a new, highly informative dataset called MrMT, which demonstrate that TasselLFANet outperforms the latest batch of lightweight networks in terms of performance, flexibility, and adaptability, achieving an F1 measure value of 94.4%, a mAP.@5 value of 96.8%, and having only 6.0M parameters. Moreover, compared with the regression-based TasselNetV3-Seg† model, our proposed model achieves superior counting performance, with a mean absolute error (MAE) of 1.80, a root mean square error (RMSE) of 2.68, and a R^2^ of 0.99. The proposed model meets the accuracy and speed requirements of the vision system in maize tassel detection. Furthermore, our proposed method is reliable and unaffected by geographical changes, providing essential technical support for computerized counting in the field.

## 1 Introduction

Maize has been a crucial agricultural crop for several decades, serving as a primary source of food, feed, fuel (ethanol), and other industrial feedstocks (Andorf et al., 2019). However, the yield of maize kernels is prone to fluctuation due to planting density issues caused by poor germination and planting errors (Gonzalez et al., 2018). Counting maize tassels is therefore an essential task in crop management and breeding, as it provides valuable information on germination rates and population densities for farmers and breeders (Wang et al., 2021). Additionally, monitoring the development of maize tassels offers a reliable foundation for observing the growth cycle of maize (Yu et al., 2013), resulting in improved production and more effective farming practices. This technique can offer practical guidance and early warning to farmers (Bai et al., 2018), making it a powerful tool for crop management.

Traditional manual observation and counting rely on labor-intensive tasks, which are not only time-consuming but also limited in sample size. Fortunately, with the rapid development of computer vision and deep learning, more advanced research methods have emerged for extracting and processing visual information from image data, providing us with better options for efficiently detecting and counting plants (Yu et al., 2017). Building on breakthroughs from the past few decades (He et al., 2015; Russakovsky et al., 2015; Vaswani et al., 2017), the field of visual recognition has entered a new era of large-scale visual representation learning. The performance of visual representation learning systems is largely influenced by three main factors: the chosen neural network architecture, the method used to train the network, and the data used for training. In the field of visual recognition, every advancement in each of these areas contributes to the overall performance improvement. Innovations in neural network architecture design have played an important role in representation learning. Convolutional neural networks (CNNs, See **Table 1** for all abbreviations in the paper.) (Chollet, 2017; Shafiq and Gu, 2022) have demonstrated high capability for learning discriminative visual representations and convincingly extended to a range of computer vision tasks such as image recognition, object detection, and semantic segmentation. Currently, related applications in object detection have gradually matured. The field is mainly divided into two-stage algorithms represented by Faster Region-based Convolutional Neural Network (Faster RCNN) (Chen and Gupta, 2017) and one-stage algorithms represented by You Only Look Once (YOLOv3) (Redmon and Farhadi, 2018). Among them, CenterNet (Duan et al., 2019), EfficientDet (Tan et al., 2020), RetinaNet (Lin et al., 2017), Yolov7-tiny (Wang et al., 2022) have achieved SOTA performance in many different fields.

**Table 1:** Summary of abbreviations.

Abb.	Full name
BN	Batch Normalization
MP	Max Pooling
REP	Re-Parameterization
GMP	Global Max Pooling
GAP	Global Average Pooling
MP-C	Max Pooling-Conv
ECA	Efficient Channel Attention
Mlt-ECA	Multi-Efficient Channel Attention
ELAN	Efficient Layer Aggregation Networks
ELAN-H	Efficient Layer Aggregation Networks-Higher
SPPCSPC	Spatial Pyramid Pooling Cross Stage Partial Connect
PANet	Path Aggregation Network
YOLO	You Only Look Once
CNN	Convolutional Neural Network
R-CNN	Region-based Convolutional Neural Network
UAV	Unmanned Aerial Vehicle
MAE	Mean Absolute Error
RMSE	Root-Mean Square Error
MAPE	Mean Absolute Percentage Error
FPS	Frames Per Second

In the field of plant science, various methods have been developed for applications such as counting trees (Li et al., 2016), fruits (Rahnemoonfar and Sheppard, 2017), wheat ears (Madec et al., 2019), and rice panicles (Wang et al., 2022a), with remarkable success. However, despite these advances, identifying maize tassels using visual techniques remains a challenging task due to the following factors:

1) The size, color, and texture of tassels vary depending on the maize cultivar and growth stage. The tassel margins are irregularly shaped, and their color may resemble that of leaves at a particular stage.
2) The illumination changes considerably under different weather conditions, leading to image degradation, wind-induced motion, camera shooting angle variations, and perspective distortion, causing differences in tassel poses.
3) Occlusion is commonly present, making it difficult to count even for human experts. The presence of a cluttered background diversifies and misleads the visual pattern of the maize tassel.

Therefore, there is an urgent need for machine learning models that can detect maize tassels in a wide variety of situations with reliable generalizability. Currently, vision-based research on grain ear numbers is mainly categorized into three groups as follows:

**Segmentation-based methods:** Segmentation-based methods are commonly used to segment plant or organs in image sequences using phenotypic features such as color and texture. The counting methods are then applied to calculate the number of ears. Several studies have been conducted to improve the accuracy and efficiency of this method for different plant species. For instance, Yu et al. (2016) utilized the spatio-temporal saliency properties of maize tassels and employed a low-rank matrix decomposition method to identify the pixel domain of the tassel. This was followed by image segmentation to extract the tassels. However, this method is only applicable to scenes where the tassels are relatively prominent against the background. Hayat et al. (2020) proposed a rice ear segmentation algorithm based on unsupervised Bayesian learning, which employs a multivariate mixture Gaussian model to represent the probability distribution of pixels. The algorithm achieved an average F1 measurement of 82.10%. Nonetheless, it may not be suitable for maize tassels due to their rich textural characteristics and different colors of different cultivars. Ma et al. (2020) developed an EarSegNet model based on semantic segmentation that integrates an encoder-decoder structure and dilated convolution. The model aims to improve the accuracy and efficiency of winter wheat ear segmentation and achieves an F1 measurement of 87.25%. Yu et al. (2022) proposed an Unmanned Aerial Vehicle (UAV) tassel image recognition algorithm based on the U-Net model. The algorithm combines lightweight and heavy extraction networks, striking a balance between accuracy and speed with a relative mean squared error RMSE of 4.4. Nevertheless, low-level noise can severely disrupt counting after phenotypic segmentation. This may result in errors accumulating and a decrease in accuracy.

**Regression-based method:** The direct counting by regression network is an alternative method for object counting. Lu et al. (2017) proposed TasselNet, a regression network that directly computes maize tassels. By modeling the local visual characteristics of field images and regressing the local counts of maize tassels, TasselNet can achieve good adaptability to in-field variations. However, this approach may not be as robust as object detectors in later growth stages. To improve counting accuracy and efficiency, the same research group proposed TasselNetV2 and TasselNetV2+ (Xiong et al., 2019; Lu and Cao, 2020). Similarly, Khaki et al. (2022) proposed WheatNet for counting wheat ears, achieving an overall prediction error of 8.7%. Liu et al. (2022) introduced IntegrateNet, a new network for maize stand counting that supervises the learning of density map and local count simultaneously. This approach balances the tradeoff between their errors, resulting in improved model performance. One disadvantage of direct counting by regression networks is that this method only provides ear counts that are as reliable as possible, making it difficult to analyze the ears phenotype accurately after counting.

**Object detection-based method:** Object detection is a popular approach for counting that involves detecting and drawing bounding boxes. This method not only provides the number of objects but also their size and location. For instance, Liu et al. (2020) used the Faster R-CNN to detect maize tassels in UAV images and achieved a detection accuracy of 89.96%. Ji et al. (2021) proposed an in-field maize tassels detection method that combines light saturation correction and Itti saliency-based systems to detect candidate regions, and false positives are removed using the LS-SVM classifier, resulting in an F1 score of 88.36%. However, object detection methods without deep learning models have relatively poor learning capabilities, which may limit their direct use in other applications. Yang S, et al. (2021) proposed an improved CenterNet that embeds location information in the feature extraction module and increases the detection accuracy to 92.4%. While the above detection methods are designed for specific cultivars in particular environments, they may not accurately distinguish tassels at early tasseling stages, making it difficult to continuously monitor maize breeding requirements. Miao et al. (2021) found that the convolutional neural network-based regression counting method had poor accuracy and high bias for plants with extreme leaf counts, while the count-by-detection method based on the Faster R-CNN object detection model achieved near-human performance for plants where all leaf tips are visible. However, the two-stage detection network used in the count-by-detection method ignores the real-time requirements of field applications. Notably, the YOLO series, another commonly used object detection method, is faster and more efficient than other methods and can meet the practical needs of plant detection and counting problems (Yang B, et al., 2021; Lyu et al., 2022; Zang et al., 2023). However, the accuracy of tassel detection still needs to be improved (Zou et al., 2020).

In recent years, embedded systems have gained significant attention as a promising approach for efficient target detection in crop growth monitoring systems, such as the identification of specific crop features. The real-time processing capabilities of embedded systems can significantly reduce the time delay between image acquisition and detection. To this end, several studies (Gajjar et al., 2021; Saddik et al., 2022) have presented intelligent plant disease diagnosis systems that integrate computer vision and machine learning techniques. However, the accuracy of such systems may be affected by image quality and lighting conditions, and they may require substantial computational resources and training time. In a similar vein, a fruit detection and counting system that leverages embedded systems and convolutional neural networks (CNNs) has been proposed (Mazzia et al., 2020; Zhang et al., 2022). Although the system can detect and count fruits in real-time, its accuracy may be influenced by factors such as fruit color, size, and shape, and it requires significant computational resources. Therefore, there is a need for vision technologies that can ensure high accuracy while being easy to implement. Specifically, there is a need to address the impact of external environmental conditions and internal growth features on object detection while reducing the computational requirements of embedded systems.

To address these challenges, this paper proposes a new lightweight neural network called TasselLFANet, which is based on the deep learning framework. The network has a faster and stronger structure and a more efficient feature integration method. We enhance the feature-learning ability of the network by using a cross-stage fusion strategy that balances the variability of different layers. Moreover, our method makes use of diverse feature representations with multiple receptive fields and introduces an innovative visual channel attention module to detect and capture features more flexibly and accurately.

Overall, the main contributions of this paper are four-fold:

1) Through a series of comparative experiments, we demonstrate that detection-based methods hold great promise for plant counting applications. Compared to regression-based methods, detection-based methods provide more comprehensive object information, such as position and size, which can inform pre- and post-processing steps.
2) We propose a novel global regression framework for object detection, named TasselLFANet, which leverages an improved attention module to address scale changes of tassels and environmental variations in complex wild-field situations. Our framework achieves high efficiency while maintaining strong performance. In addition, our model has a small number of parameters, making it suitable for real-time detection in embedded systems.
3) We introduce MrMT (Multi-regional Maize Tassels), a highly informative, spatially and temporally continuous dataset containing 1968 images and 96434 corresponding bounding box annotations. This large-scale, high-cost dataset is designed to provide researchers with a more convenient and detailed resource for agricultural research, including crop growth stage detection.
4) Based on the evaluation of the MrMT dataset, we demonstrate that our proposed method outperforms the latest batch of high-performance lightweight networks for object detection. Furthermore, our method surpasses the state-of-the-art TasselNetV3-Seg† model in plant counting performance when compared to regression network-based counting methods.

## 2 Materials and methods

### 2.1 Image collection and annotation

**Figure 1:**
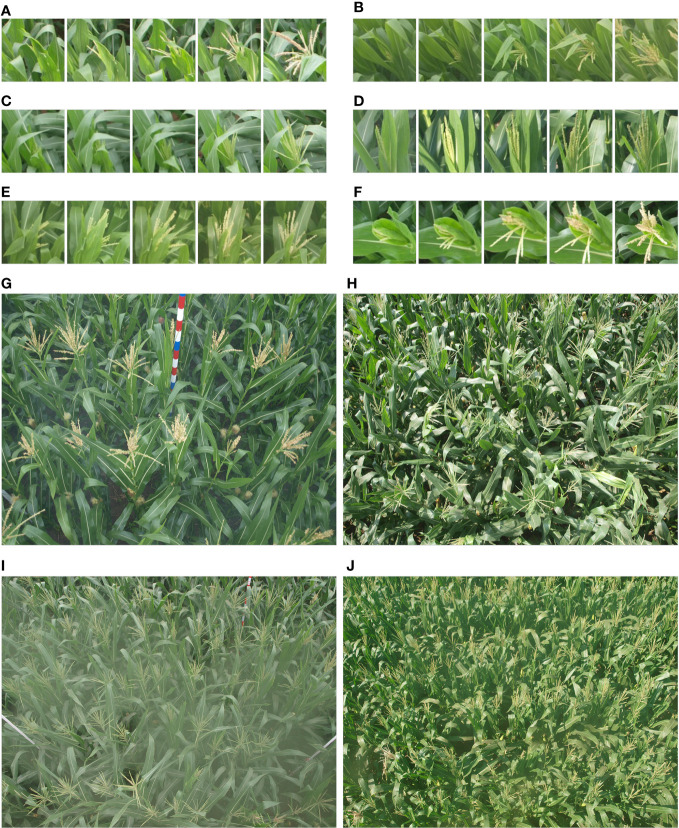
Example of field imaging of maize tassels in the MrMT dataset. **(A–F)** present images of tassels from tasseling stage to flowering stage in multiple time sequences; **(G–J)** are example images in different scenarios, respectively from Hebei2012, Shandong2011_1, Shandong2010_2, Henan2012, it can be observed that the growth environment of maize is complex and is challenging for automatic operations.

We developed an automated ground-based observation system that captures farmland images continuously every day. The digital camera captures eight images per day, one every hour from 9:00 to 16:00. The system was installed in experimental fields located in Tai’an of Shandong province, Zhengzhou of Henan province, and Gucheng of Hebei province, China, as described in Yu et al. (2013). The experimental fields grew various cultivars of maize. The MrMT (Multi-regional Maize Tassels) dataset was collected, containing 12 independent image sequences from the tasseling stage to the flowering stage, totaling 1968 field images, as listed in **Table 2**. To capture subtle changes, the dataset covers various scales and environments through continuous sampling, which helps to learn network representations based on quantitative data. The MrMT dataset enriches the existing dataset of maize tassels images with more timepoints, as shown in **Figure 1**. The dataset includes images of maize tassels in different scenarios, such as tasseling stage to flowering stage in multiple time sequences (**Figures 1A–F**), and example images from different locations (**Figures 1G–J**), including Hebei2012, Shandong2011_1, Shandong2010_2, and Henan2012. These images highlight the complexity of the maize growth environment, making it challenging for automatic operations.

**Table 2:** Multi-regional Maize Tassels (MrMT dataset).

Image Sequence	Total Size	Train Size	Valid Size	Test Size
*Shandong2010_1*	116	71	30	15
*Shandong2010_2*	117	69	30	18
*Shandong2011_1*	140	79	30	31
*Shandong2011_2*	126	61	30	35
*Shandong2012_2013*	55	–	–	55
*Henan2010*	221	140	60	21
*Henan2011*	237	140	60	37
*Henan2012*	227	140	60	27
*Henan2014*	240	140	60	40
*Hebei2010*	225	140	60	25
*Hebei2012*	220	140	60	20
*Hebei2014*	44	–	–	44

**Figure 2:**
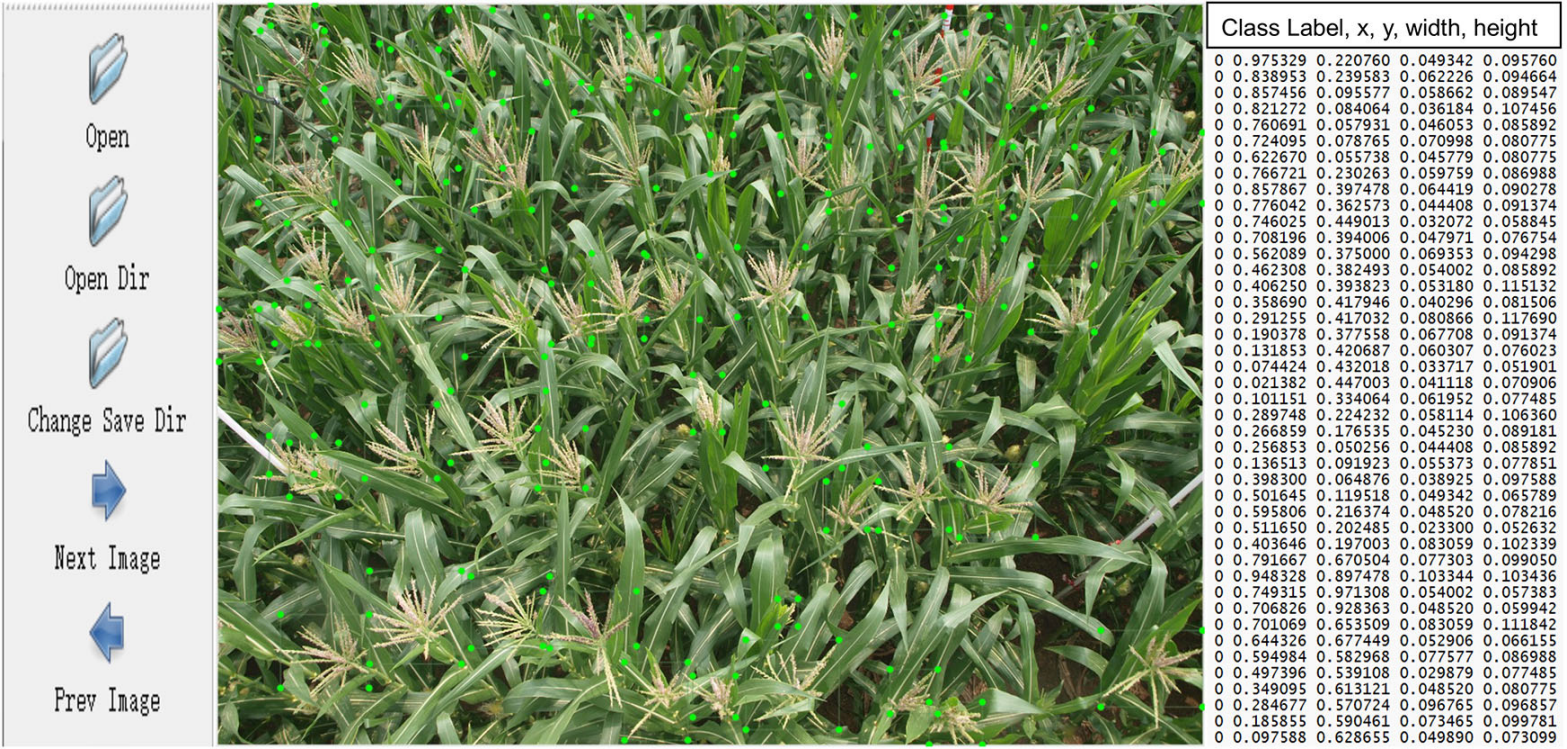
Box-level labeling for MrMT dataset.

In accordance with standard annotation paradigms, box-level labeling was manually performed for each maize tassel using the open-source tool Labelimg (Tzutalin, 2022). An example of such labeling is shown in **Figure 2**. While this work was both expensive and time-consuming, it proved to be meaningful and valuable, resulting in the annotation of a total of 96,434 maize tassels. It is important to note that these labels may contain some level of noise, which can increase with the amount of data and make training the model more challenging. However, our proposed method has demonstrated excellent noise suppression capabilities, as we will show in later sections, and is also able to adapt to the domain of the MrMT dataset.

### 2.2 Lightweight feature aggregation network (TasslLFANet)

**Figure 3:**
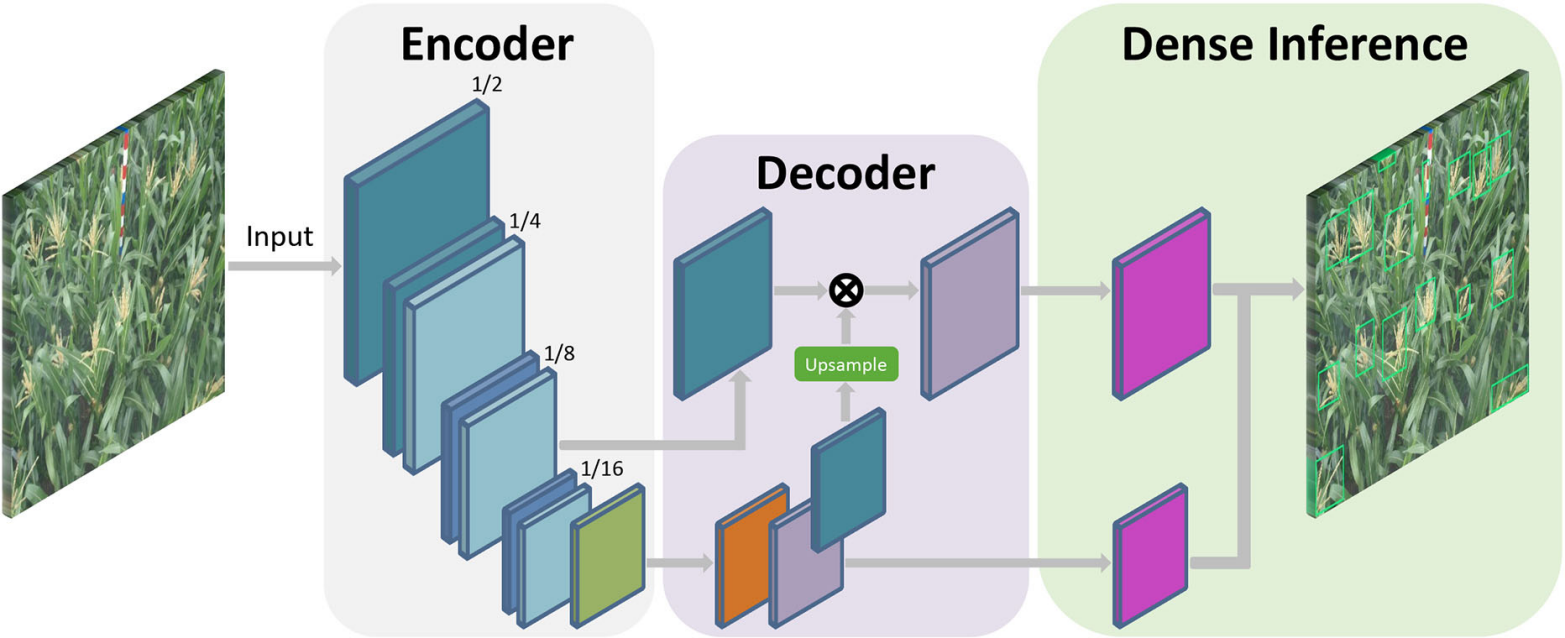
TasselLFANet global regression framework ( ⊗ indicates Concat operation).

Our proposed method is a one-step global regression framework that directly maps image pixels to bounding boxes, coordinates, and classification scores. The network architecture is designed to be simpler and more efficient, allowing for real-time performance. Our method employs binary cross-entropy loss as the supervisory signal and includes a box regression branch that predicts four coordinates for each box, along with an objectness score. The objectness score is equal to 1 if the anchor box overlaps with the ground-truth box more than any other anchor box. The overall architecture is depicted in **Figure 3** and consists of Encoder, Decoder, and Dense Inference components, which we will explain in detail in the following sections.

#### 2.2.1 Efficiency aggregation encoder

**Figure 4:**
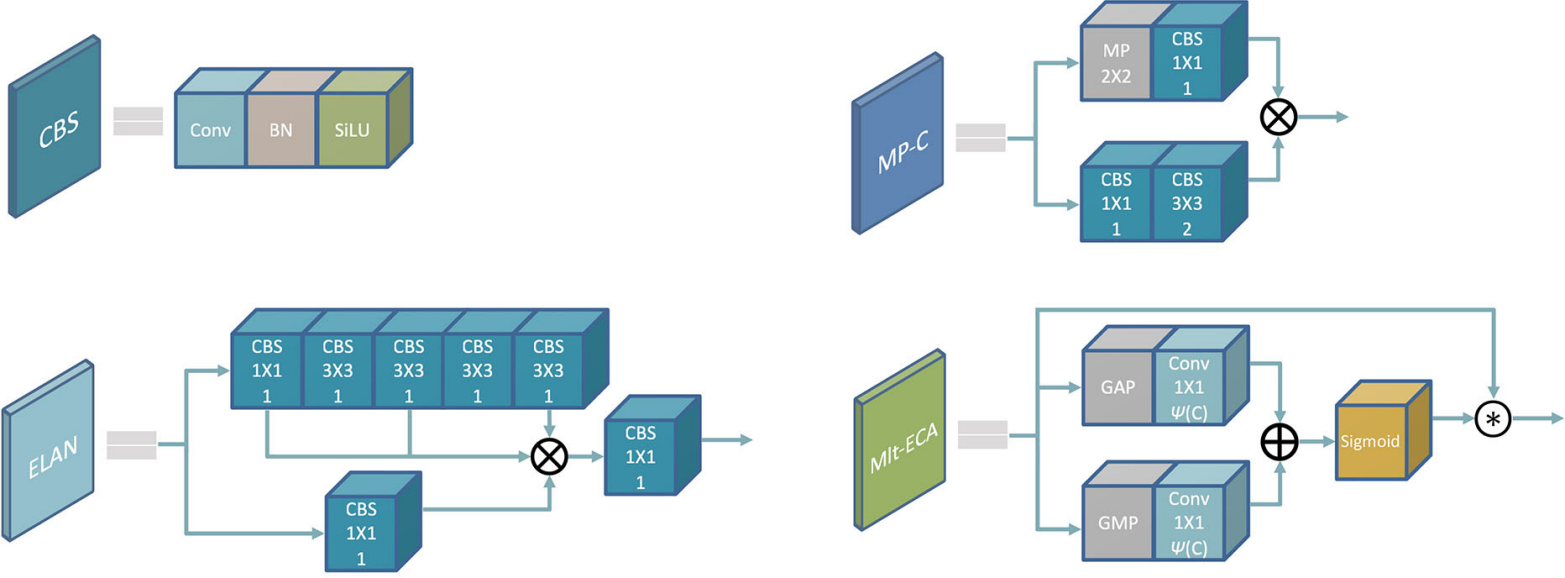
Encoder composition module: (CBS/Conv: K × K, S; MP: S × S. K × K represents the convolution kernel size, S represents the step size).

We use the convolution, batch normalization, and SiLU activation functions as basic components of the model. Efficient Layer Aggregation Network (ELAN) (Wang et al., 2022b) and Max Pooling-Conv (MP-C) modules constitute an Encoder for feature extraction. As shown in **Figure 4**, an image of size of H × W × 3 is taken as input, the feature maps are performed by multi-dimensional aggregation, and the feature maps are output in two-fold down sampling manner. The encoder can then be divided into four stages, each stage outputting 32, 64, 128, and 256 channels respectively, and the mapping information is used as the encoding set for the input image. ELAN is our important means of feature encoding. Through cross-channel information interaction, it aggregates different feature layers into the output feature map. Such a jump-level structure integrates multi-layer outputs, which enhances the expressiveness and adaptability of the network. By the way, the MP-C down sampling method that combines pooling and convolution is adopted to reduce the image information lost after pooling. Combined with convolution, superficial information from shallow layers and semantic information from deep layers are aggregated to reduce feature dimensionality while retaining useful information, avoiding overfitting to some extent. Finally, we also add an attention module Multi-Efficient Channel Attention (Mlt-ECA), which improves the model’s performance. Mlt-ECA is our innovation based on ECA (Wang Q, et al., 2020), which will be described next.

#### 2.2.2 Multi-branch decoder

**Figure 5:**
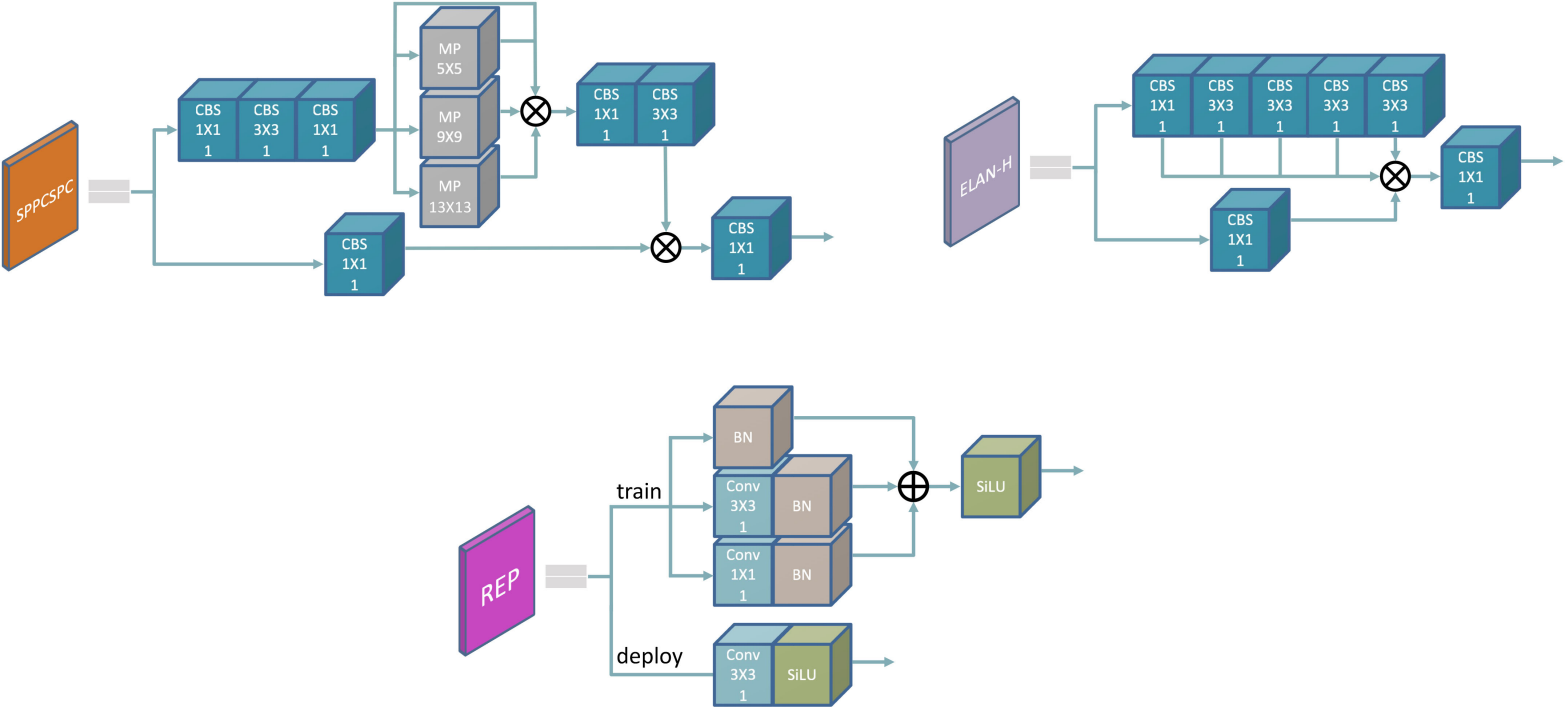
Decoder components and REP module ( ⊗ means add operation).

Multi-Branch Decoder, as a concise and effective decoder, utilizes the correlation between feature maps to enhance feature reuse through multi-dimensional interaction and aggregate rich information flows to enhance feature hierarchy. In order to better separate context information and obtain multi-level receptive fields, we embed the Spatial Pyramid Pooling Cross Stage Partial Connect (SPPCSPC) module at the connection between Encoder and Decoder. The SPPCSPC module uses group convolution, which is efficient for the model, where cross-stage feature fusion strategy and truncated gradient flow have been adopted to improve the variability of learned features within different layers (Wang et al., 2020), thereby obtaining aggregated information at different scales and enriching the expressive power of feature maps. To cope with the scale change and perspective transformation in the image, we merge the output feature map of the third stage in the Encoder, and increase the spatial dimension between the cascades by using the nearest neighbor upsampling on the Decoder output layer, so as to obtain feature maps that have the same size to the Encoder output. The 1 × 1 convolution is used to map the feature map to the same channel dimension to achieve concat splicing, and the spliced feature map is used as the second branch of the Decoder. The feature information is output after being remapped by the module Efficient Layer Aggregation Networks-Higher (ELAN-H). ELAN-H is an extension of the ELAN module that further enhances network learning capabilities without destroying the original gradient path. Two branches are used for detection on images with different scale transformations. They augment each other and adjust dynamically to improve the detection accuracy for object with multi-scales. **Figure 5** shows the components of the Multi-Branch Decoder.

#### 2.2.3 Dense inference

The Dense Inference module aims to merge the sub-images detection results from the information obtained at different stages, and further overlay the encoded information onto the original feature image. In real-world scenarios, training resources are generally relatively abundant, and people are more concerned about the inference overhead and performance, which is why we choose to use the Re-Parameterization (REP) module (Ding et al., 2021). The structure of REP is shown in **Figure 5**, which is based on a structural reparameterization implementation. Recently, the new concept of reparameterization has become an important topic in network training and object detection (Ding et al., 2022; Hu et al., 2022). During training, the network retains different receptive fields through multi-gradient flow path mapping features to enrich encoding information for better performance gain. During inference, all network layers are transformed into 3 × 3 convolutions using an Op-fusion strategy to achieve the goal of not increasing computational load, which is useful for network deployment and acceleration.

#### 2.2.4 Mlt-ECA attention module

In recent years, attention mechanisms have been proven to have broad prospects for improving the performance of CNNs (Niu et al., 2021; Guo et al., 2022). They tend to be less computationally expensive than CNNs due to their lower structural complexity and number of parameters. Convolution operations are now an important means of extracting features of objects, and attention mechanism can modify the extracted features while preserving valuable information. This allows the model to ignore the background and pay more attention to the information it needs. The ECA method (Wang CY, et al., 2020) proposes a local cross-channel interaction strategy without dimensionality reduction. This strategy uses an adaptive one-dimensional convolutional kernel size method to determine the coverage of local cross-channel interactions. Based on the ECA step, we add another branch that considers building the dependencies between channel information in the module’s internal feature map. As shown in **Figure 4**, this branch works similarly to the original ECA branch map, replacing average pooling with max pooling. Channel weights for both the average pooling branch and the global pooling branch are generated by performing a 1D convolution with a kernel size of k , where k is adaptively determined by mapping the channel dimension C and can be expressed as Eq.1. The features of the two channels are then aggregated, and finally output after the Sigmoid function feature recalibration.


(1) $$k=\text{Ψ}\left(C\right)+{\left|\frac{\log_{2}C}{\gamma }+\frac{\beta }{\gamma }\right|}_{odd}$$

Among them, k represents the size of the convolution kernel, C is the number of channels, odd indicates that k is odd, and γ and β are set to 2 and 1 respectively in the experiment to change the ratio between C and the convolution kernel. To easily distinguish the improved attention mechanism from the original ECA attention module, our proposed module is called Mlt-ECA.

Its working principle can be described by the following formula:


(2) $$Ce\left(E\right)=\sigma \left(CL_{k}\left(E_{AP}^{C}\right)+CL_{k}\left(E_{MP}^{C}\right)\right)$$

(3) ME=Ce(E)⊛E

where $E_{AP}^{C}$ and $E_{MP}^{C}$ represent the features of average pooling and maximum pooling respectively, $CL_{k}$ refers to the one-dimensional convolution of each pixel with its nearest neighbor k pixels, σ represents the Sigmoid function, and ⊛  represents element-wise multiplication. ME denotes the output feature map obtained by Mlt-ECA attention.

#### 2.2.5 Loss function

In deep learning, the loss function plays a crucial role in adjusting the weight of the neural network during backpropagation. In our work, we propose two loss functions to guide the bounding box regression. These functions are designed to calculate the error between the predicted values and the ground-truth values during the forward propagation stage, and are used to optimize the network parameters in the training process.

##### 2.2.5.1 Localization loss

We use Loc to compute the localization difference between the prediction box and the ground-truth box for each image. Now let $b_{pd}$ , $b_{gt}$ be the center of the prediction bounding box and the ground-truth bounding box, respectively, then


(4) $$Loc=IoU-\frac{\rho^{2}\left(b_{pd},b_{gt}\right)}{c^{2}}-\alpha \nu$$

where ρ is the Euclidean distance between the two centers, c is the diagonal length of the minimum enclosing rectangle of the prediction box and the ground-truth box, and IoU represents the intersection ratio between the ground-truth box and the prediction box. Among them, ν is used to measure aspect ratio similarity, and α is the influencing factor of ν , which are defined as:


(5) $$\nu =\frac{4}{\pi^{2}}{\left(arctan\frac{w_{pd}}{h_{pd}}-arctan\frac{w_{gt}}{h_{gt}}\right)}^{2}$$

(6) $$\alpha =\frac{\nu }{1-IoU+\nu }$$

where $w_{pd}$ and $w_{gt}$ are the widths of prediction boxes and ground-truth box, respectively; $h_{pd}$ and $h_{gt}$ are the heights of prediction boxes and ground-truth box, respectively.

##### 2.2.5.2 Confidence loss and classification loss

Confidence Loss $L_{xj}^{obj}$ and Classification Loss $L_{xj}^{cls}$ use the binary cross-entropy function BCEWithLogitsLoss as supervision to measure the cross-entropy between the target and the output. As for a two-category task, for a sample, it is assumed that the predicted probability of one class is p , and the other class is 1−p .


(7) $$P_{i}=\{\begin{array}{c} p,\;if\;y=1\\ 1-p,\;otherwise \end{array}$$

$P_{i}$ represents the probability that sample i is predicted to be a positive sample, then the loss can be defined as:


(8) $$Lxj=-\frac{1}{N}\sum_{i}\left[y_{i}\ln P_{i}+\left(1-y_{i}\right)\ln\left(1-P_{i}\right)\right]$$

$y_{i}$ represents the label of sample i, the $y_{i}$ is set to 1 when i belongs to the positive class, the negative class is 0, and N represents the total number of samples.

Then, we combine the three losses to get the final loss of TasselLFANet, namely


(9) $$L_{fan}=Loc+Lxj$$

where $Lxj=L_{xj}^{obj}+L_{xj}^{cls}$

## 3 Experiments and discussions

### 3.1 Implementation details

In this study, we used a deep learning approach to detect and count maize tassels. Our training dataset consisted of 1120 images randomly selected from the publicly available MrMT dataset. We reserved 480 and 368 images as validation and test sets, respectively. All experiments were conducted on a deep learning framework implemented with PyTorch 1.8 and CUDA 9.0, and executed on an Nvidia Quadro P5000 GPU with 16G of memory. We did not rely on pre-trained model weights during transfer learning to ensure that our model’s performance reflected its true potential (Mesnil et al., 2012; He et al., 2019). Given the high resolution of the images in our dataset, we resized them to 640 x 640 pixels. We used the cosine function to schedule the learning rate, which started at 0.01. The training was performed with stochastic gradient descent (SGD) optimizer with a momentum of 0.937, and lasted for 100 epochs. To prevent overfitting and enhance the robustness of our model, we applied various data augmentation techniques, including color distortion, random translation, random flipping, random scaling, and random stitching.

### 3.2 Comparison with object detection models

#### 3.2.1 Evaluation metrics

The evaluation metrics of the TasselLFANet model compared to other models are mainly based on precision ( P ), recall ( R ), mean precision ( mAP ) and F1−measure , where TP , FP and FN are the number of true positives, false positives and false negatives, respectively. n is 1 because there is only one category (maize tassel) in the data. mAP@0.5 represents the average mAP at an IOU threshold of 0.5. mAP@0.5:0.95 represents the average of mAP at different IOU thresholds (from 0.5 to 0.95 in step of 0.05).


(10) $$P=\frac{TP}{TP+FP}$$

(11) $$R=\frac{TP}{TP+FN}$$

(12) $$mAP=\frac{\sum_{1}^{n}\int_{0}^{1}P\left(R\right)d\left(R\right)}{n}$$

(13) $$IOU=\frac{Area\;of\;Overlap}{Area\;of\;Union}$$

(14) $$F1-measure=2\frac{PR}{P+R}\in \left[0,1\right]$$

#### 3.2.2 Ablation study

Since attention is a plug-and-play module, we examine the impact of adding attention at various locations within the model architecture, conducting three different ablation implementations of ECA and Mlt-ECA. These implementations allow us to assess the benefits of integrating attention modules within the Encoder and after the Decoder has decoded the output layer. Our experimental results, as summarized in **Table 3**, demonstrate that Mlt-ECA consistently outperforms ECA when added in different positions. Moreover, we observe that the effect of adding after Decoder decoding the output layer is better than adding at two positions, and the highest performance is achieved when adding Mlt-ECA after the Encoder. Notably, our modified TasselLFANet with Mlt-ECA achieves an F1 score that is 0.4% higher than the original TasselLFANet, with P increasing by 0.6% to 0.946 and R increasing by 0.4% to 0.942. These findings indicate that Mlt-ECA is more robust and effective in suppressing background information, enabling the model to focus on foreground features and enhance high-level semantic understanding.

**Table 3:** Ablation experiments of ECA attention and Mlt-ECA attention.

Model	Encoder	Decoder	P	R	F1	mAP@.5	mAP@.5:.95	Param
*TasselLFANet-original*	–	–	0.940	0.938	0.938	**0.968**	0.543	6.0M
*ECA*	✔	✔	0.942	0.934	0.938	0.963	0.524	6.0M
*MltECA*			0.945	0.940	0.942	**0.968**	0.54	6.0M
*ECA*	–	✔	0.941	0.936	0.938	0.964	0.526	6.0M
*MltECA*			**0.948**	0.940	**0.944**	**0.968**	0.541	6.0M
*ECA*	✔	–	**0.948**	0.938	0.943	**0.968**	0.545	6.0M
*MltECA*			0.946	**0.942**	**0.944**	**0.968**	**0.546**	6.0M

“✔” means joining the corresponding module and the best performance is in boldface.

#### 3.2.3 Comparing experimental results

Based on the MrMT dataset, we compare TasselLFANet with six state-of-the-art lightweight object detection methods according to the actual application needs, including SSD (Liu et al., 2016), RetinaNet (Lin et al., 2017), CenterNet (Duan et al., 2019), EfficientDet (Tan et al., 2020), Yolox-nano (Ge et al., 2021b) and Yolov7-tiny (Wang et al., 2022a). We train and test these networks using the same training and test sets. A comparison of each method is shown in the **Table 4**.

**Table 4:** Comparison of evaluation indicators of different models.

Model	Test Size	Backbone	P	R	F1	mAP@.5	mAP@.5:.95	Param
*SSD*	300	VGG-16	0.785	0.517	0.623	0.711	0.241	90.6M
*RetinaNet*	600	ResNet-50	0.914	0.719	0.805	0.828	0.376	138.0M
*CenterNet*	640	ResNet-50	0.874	0.928	0.900	0.902	0.452	124.0M
*EfficientDet*	512	EfficientNet-B0	0.914	0.712	0.800	0.825	0.444	15.1M
*Yolox-nano*	640	CSPDarknet-53	0.852	0.872	0.862	0.894	0.437	**3.7M**
*Yolov7-tiny*	640	E-ELAN	0.921	0.878	0.899	0.938	0.434	11.7M
*TasselLFANet*	640	ELAN	**0.946**	**0.942**	**0.944**	**0.968**	**0.546**	6.0M

The best performance is in boldface.

Obviously, our proposed model outperforms the other methods in different dimensions, and the model has fewer parameters. In general, as R increases, P decreases accordingly, and our model has a more robust trade-off. SSD, as a traditional one-stage object detection model without bounding boxes generation, uses smaller convolutional filters for dense sampling, which enables simple end-to-end training even on low-resolution input images. However, low feature layers and low number of convolutions will lead to insufficient extraction of shallow feature map information, making it difficult to meet the detection of small-scale tassels. RetinaNet and EfficientDet are good anchor-based object detection models that require extraction of candidate anchor points before making predictions. But they are also likely to miss small and dense objects due to background interference, large overlap of maize tassels and complex growth environment. The YOLO series of object detection algorithms are famous and well-balanced in speed and accuracy. Yolox-nano and Yolov7-tiny are state-of-the-art detection models that use multi-scale information in combination with the path aggregation network PANet (Liu S, et al., 2018) to enhance the feature hierarchy, which significantly improves the detection accuracy, while at the same time it is clear that the parameter size is small. CenterNet employs keypoint estimation to find center points and regress other target attributes. This detection method is novel and specific, and has a higher recall rate. It should be noted that when the IOU increases above 0.5, TasselLFANet has a higher mAP compared to the other methods, 9.4% higher than CenterNet, the second-ranked model, indicating that the model has more accurate positioning performance. Therefore, our model can achieve the best results.

#### 3.2.4 Maize tassels detection at different resolutions

**Figure 6:**
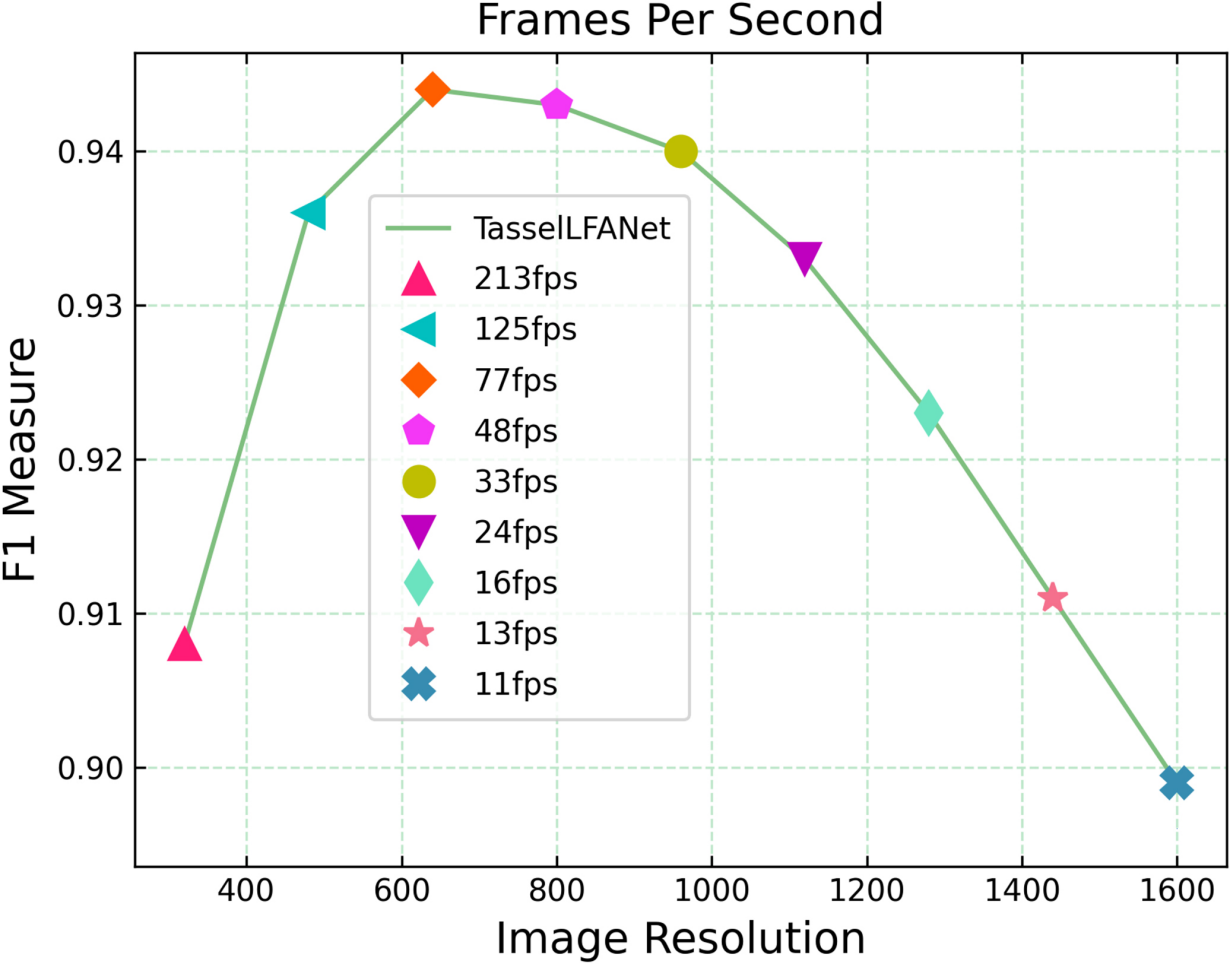
Relationship between image resolution, F1-measure and frames per second (FPS) in image scaling.

Next, we explore the relationship between image resolution, F1-measure and frames per second (FPS) in image scaling. Speeds are measured on a desktop computer with a single Nvidia Quadro P5000 GPU (16G) at different resolutions and FPS averaged over 100 forward passes. Taking the image resolution of 640 × 640 as the benchmark, the results are shown in **Figure 6**. It can be seen that the F1-measure value shows a certain attenuation tendency as the image resolution increases or decreases. In general, increasing the image resolution of the input layer may potentially result in a higher fine-grained feature representation. The higher the resolution, the better the accuracy below a certain threshold. Beyond this threshold, the improvement in accuracy begins to decline. We believe that the reason for the attenuation is that the scale distribution of samples in our dataset tends towards medium-resolution and the gain from increasing resolution tends towards saturation. At the same time, high-resolution images increase the amount of computation, and image resolution is negatively correlated with FPS.

Please note that different devices and methods have been used to capture field images, so there may be differences in resolution. Therefore, it is important that the proposed method is robust to images with different resolutions. As shown in **Figure 6**, when the image resolution is reduced to 480 and 320, the F1-measure obtains high-efficiency fps of 125 and 213 respectively, showing high performance of 0.936 and 0.908. Based on this performance, we introduce a high-efficiency model TasselLFANet-HE with a resolution of 480 × 480 pixels. Its overall performance is shown in **Table 5**.

**Table 5:** Performance comparison between TasselLFANet-HE and TasselLFANet.

Method	Test Size	P	R	F1	mAP@.5	mAP@.5:.95	FPS
*TasselLFANet*	640	0.946	**0.942**	**0.944**	**0.968**	**0.546**	77
*TasselLFANet-HE*	480	**0.947**	0.926	0.936	0.962	0.518	**125**

The best performance is in boldface.

#### 3.2.5 Overall performance evaluation

**Figure 7:**
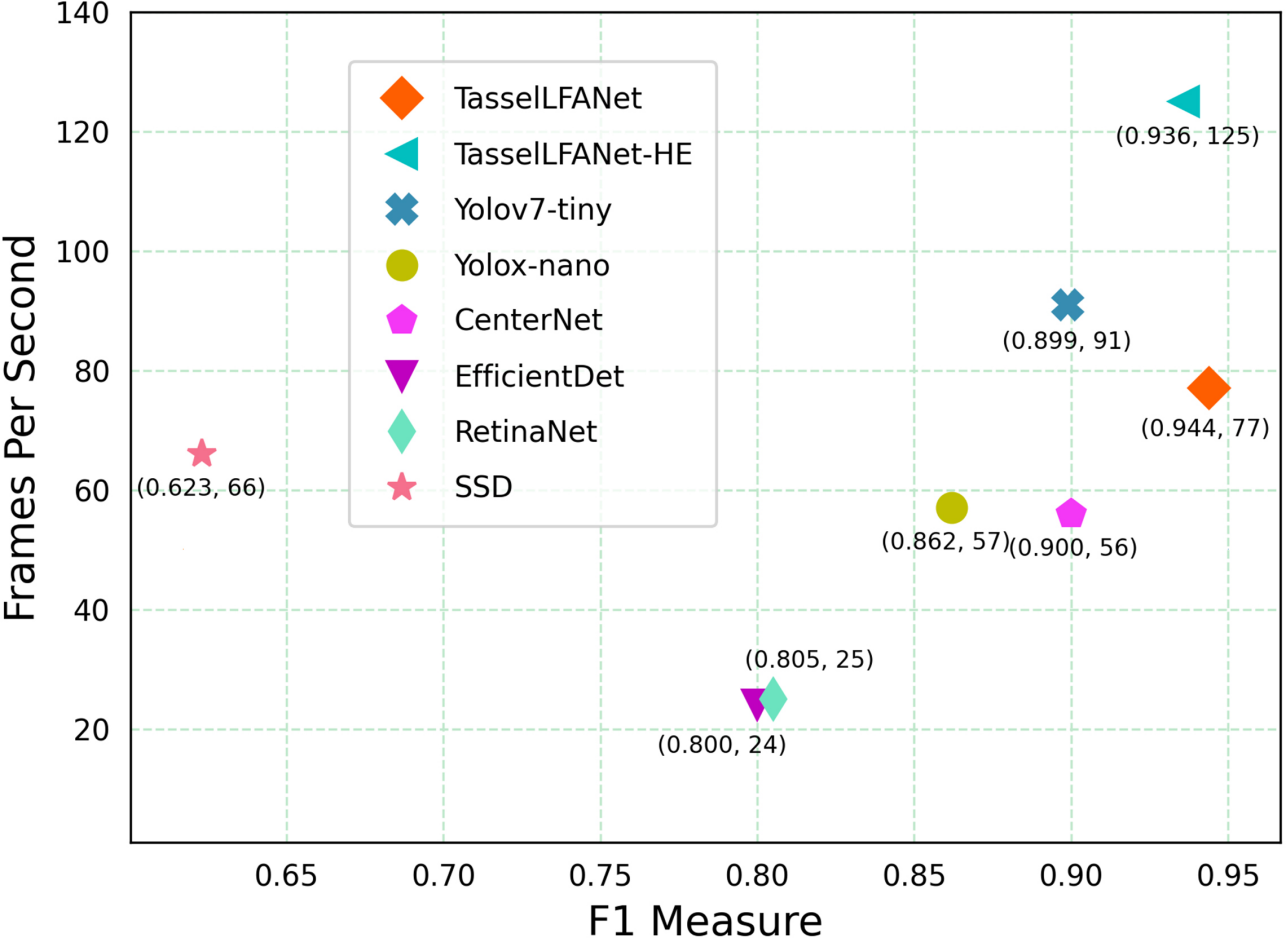
Performance and speed comparison of different models.

To further evaluate the effectiveness of the model’s overall performance, we compared TasselLFANet and TasselLFANet-HE with other detection methods. **Figure 7** shows a comparison of each model with respect to F1-measure and running speed. In brief, the TasselLFANet series model outperforms all other methods. Yolov7-tiny is faster than TasselLFANet. Although TasselLFANet has a lower parameter volume than Yolov7-tiny, TasselLFANet is less efficient than Yolov7-tiny due to feature aggregation reusability, which is to some extent expected. In addition, TasselLFANet-HE far surpasses other methods in speed, and achieves high performance that only TasselLFANet surpasses. In the future, even more speeds can be achieved with more advanced hardware devices.

#### 3.2.6 Domain adaptation comparison

**Figure 8:**
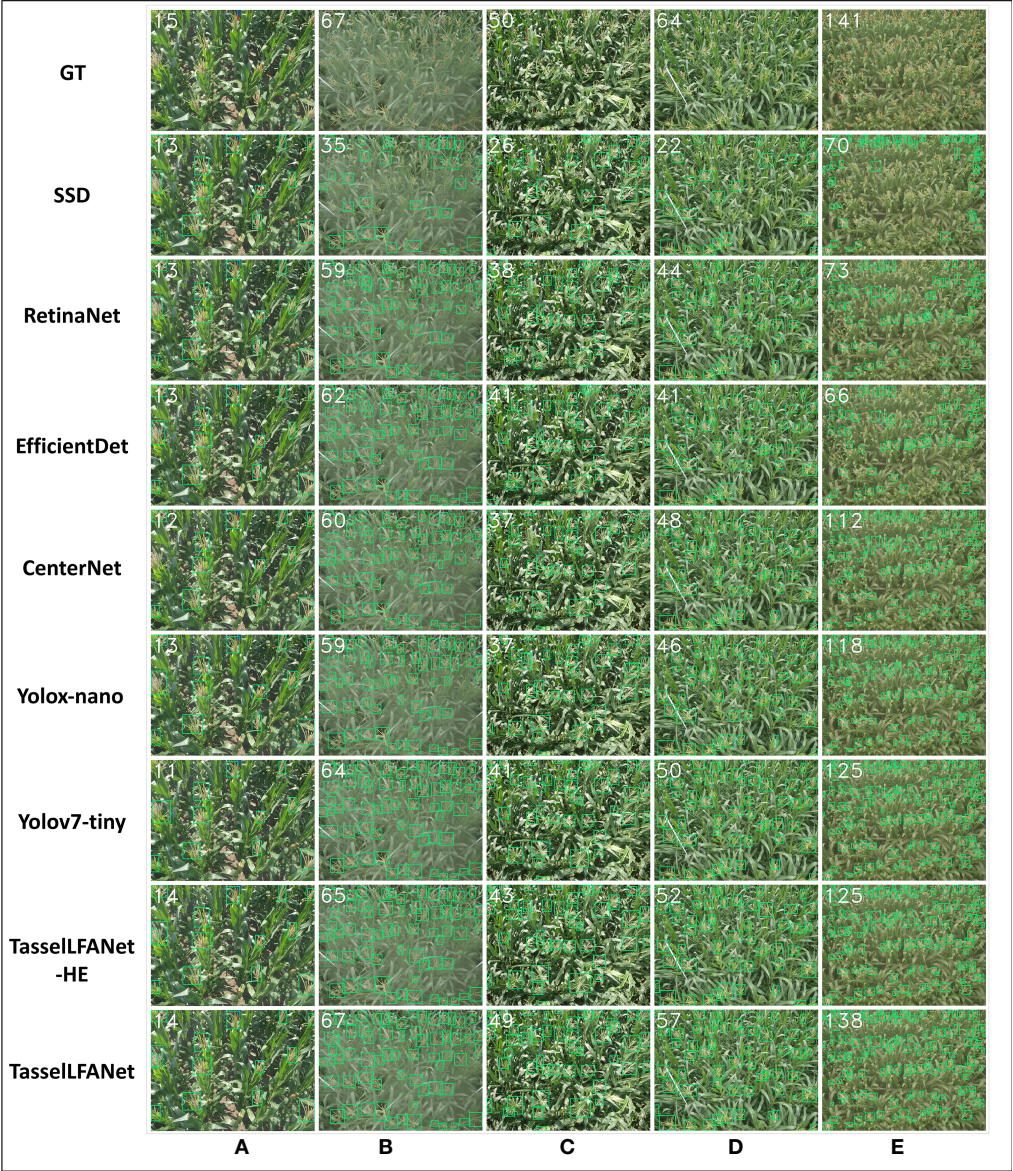
MrMT data set visualization results. The representative plant sample images for testing the domain adaptation of each model. GT means ground-truth count. **(A)** sparse tassel; **(B)** blurred image; **(C)** strong illumination; **(D)** severe occlusion; **(E)** dense object..

From the MrMT dataset, we selected a number of representative plant sample images such as sparse tassel(a), blurred image (b), strong illumination (c), severe occlusion (d), dense object (e), to test the domain adaptation of each model. As shown in **Figure 8**, the visualization results further confirm the strong adversariality of our method in the face of scale transformation and environmental disturbance. The green box in the figure is the detection result, and the white numbers in the upper left corner of each image are the counting results. The first row in the Figure is the ground-truth result, and the rest rows are the prediction results of the eight models.

**Figure 8A** shows that all models can produce reasonable approximations to ground-truth counts when individual tassels are clearly visible. However, under the complex environment shown in **Figures 8B**, **C**, especially in **Figure 8C**, the image noise increases with the increasing external illumination, and the prediction errors of other models increases. In **Figure 8D**, the tassels are occluded by the background, and although our algorithm still performs relatively well, it is difficult to provide a targeted solution for detection. In this regard, we propose to improve the data sampling method, guide the model to learn the internal characteristics contained in the dataset with a tendency, and use a more effective strategy for positive and negative sample distribution (Zhu et al., 2020; Ge et al., 2021a; Li et al., 2022). As shown in **Figure 8E**, the SSD, RetinaNet and EfficientDet algorithms are weak in representation for shallow feature maps, and these algorithms are not robust when tassels are dense and small in size. Significantly, our model performs well in this scene, with improvements that allow for more subtle image capture of tassels.

### 3.3 Comparing against the state-of-the-art counting method

#### 3.3.1 Evaluation metrics

Four metrics including mean absolute error (MAE), root-mean square error (RMSE), mean absolute percentage error (MAPE), and the coefficient of determination (R^2^) were used to evaluate the agreement between the predicted and ground-truth values. To be more specific, these metrics are computed by Eq. (15-18).


(15) $$MAE=\frac{1}{n}\sum_{i=1}^{n}\left|{\hat{y}}_{i}-y_{i}\right|$$

(16) $$RMSE=\sqrt{\frac{1}{n}\sum_{i=1}^{n}{\left(y_{i}-{\hat{y}}_{i}\right)}^{2}}$$

(17) $$MAPE=\frac{1}{n}\sum_{i=1}^{n}\left|\frac{{\hat{y}}_{i}-y_{i}}{y_{i}}\right|\;\;$$

(18) $$R^{2}=1-\frac{\sum_{i=1}^{n}{\left(y_{i}-{\hat{y}}_{i}\right)}^{2}}{\sum_{i=1}^{n}{\left(y_{i}-{\bar{y}}_{i}\right)}^{2}}$$

To further demonstrate the superiority of our model, we compared all above-mentioned object detection methods with the state-of-the-art counting method, local density regression-based TasselNetV3-Seg† (Lu et al., 2021). The test images are 181 pictures randomly selected from the MrMT test set. We also report results on the number of model parameters and frames per second (FPS), as indicators of computational complexity. **Table 6** shows the counting performance of all models related to the evaluation metrics.

**Table 6:** Counting performance comparison of different models.

Model	Param	FPS	MAE	RMSE	MAPE	R^2^
*SSD*	90.6M	66	9.04	11.99	34.3%	0.7477
*EfficientDet*	15.1M	24	4.22	6.65	19.8%	0.9225
*RetinaNet*	138.0M	25	4.04	6.42	19.7%	0.9278
*TasselNetV3-Seg†*	7.5M	36	3.75	4.68	27.5%	0.9632
*Yolox-nano*	3.7M	57	4.02	5.15	23.2%	0.9534
*CenterNet*	124.0M	56	2.87	3.85	17.8%	0.9740
*Yolov7-tiny*	11.7M	91	3.10	4.14	17.3%	0.9699
*TasselLFANet-HE*	6.0M	**125**	2.70	3.76	14.3%	0.9751
*TasselLFANet*	6.0M	77	**1.80**	**2.68**	**9.2%**	**0.9903**

The best performance is in boldface.

#### 3.3.2 Performance comparison

Numerical comparisons in **Table 6** show that our proposed TasselLFANet series method has the best overall counting performance and leads TasselNetV3-Seg† in an all-round way. As the most advanced object detection algorithm, Yolov7-tiny is second only to TasselLFANet in terms of comprehensive performance. TasselNetV3-Seg† is less efficient than Yolox-nano, and Yolox-nano has smaller parameters, because deep convolutions make poorer use of computational units than standard convolutions.

#### 3.3.3 Coefficients of determination between different models and manual counting

**Figure 9:**
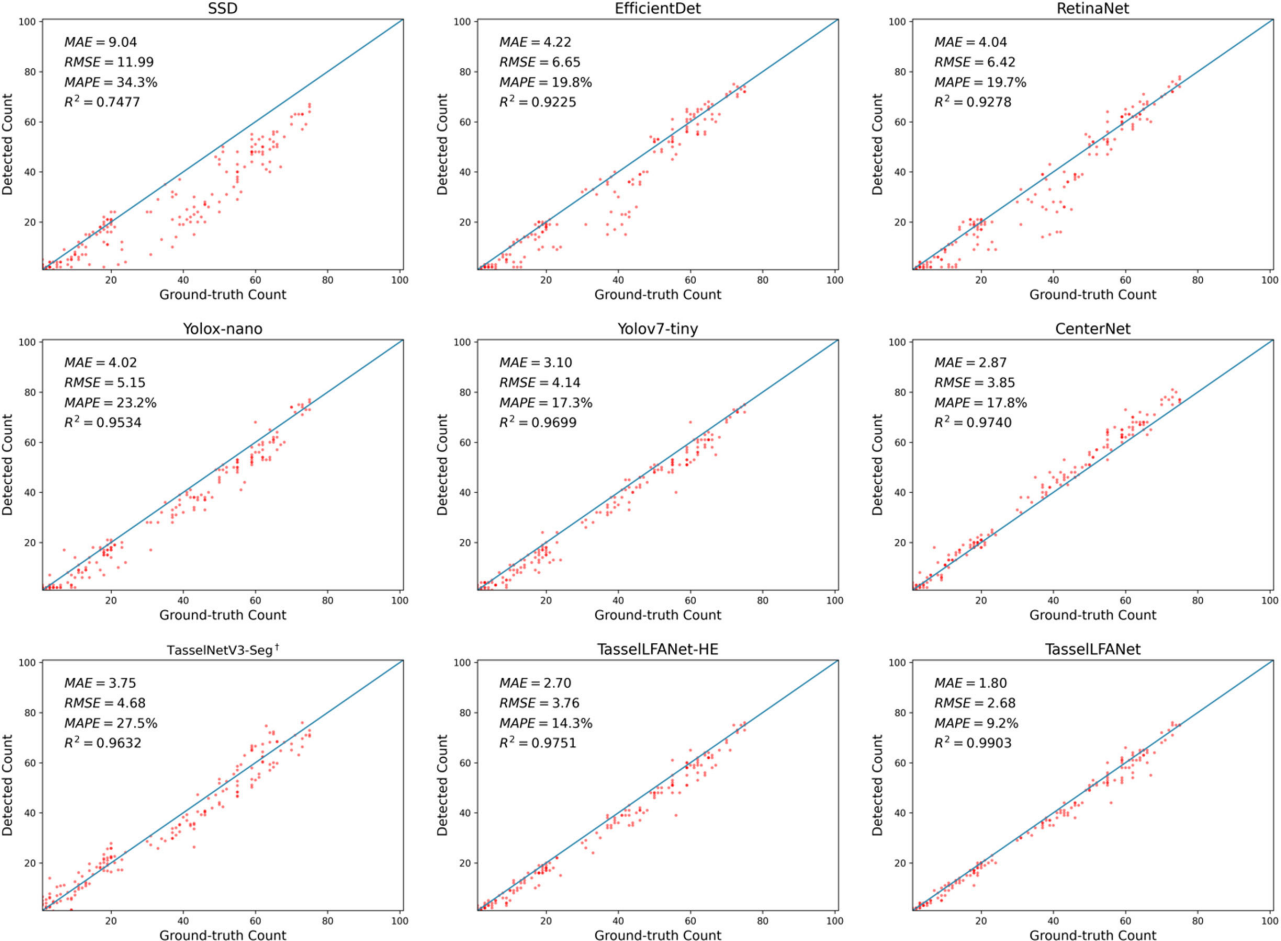
Scatter plots of the detected count by different models versus their ground-truth count.

We plot the linear regression relationship between manual counts and experimental algorithms on the MrMT dataset as shown in **Figure 9**. Presented by scatter diagram distribution, it shows that our model is able to capture changes with better robustness and generalization. The key lies in the dynamic interaction and adaptive adjustment of multiple branches of aggregate information flow, improving the performance of the model in sparse and dense scenes. Yet, the issue of occlusion has been a hot area of research in detection methods. If there are a lot of maize tassels in the image, many predicted bounding boxes will be filtered and underestimated by the non-maximum suppression of the detector. In contrast, TasselNetV3-Seg† can capture additional global information. Even though, our model achieves superior performance compared to TasselNetV3-Seg† which has large errors around the counting baseline.

## 4 Discussion

**Figure 10:**
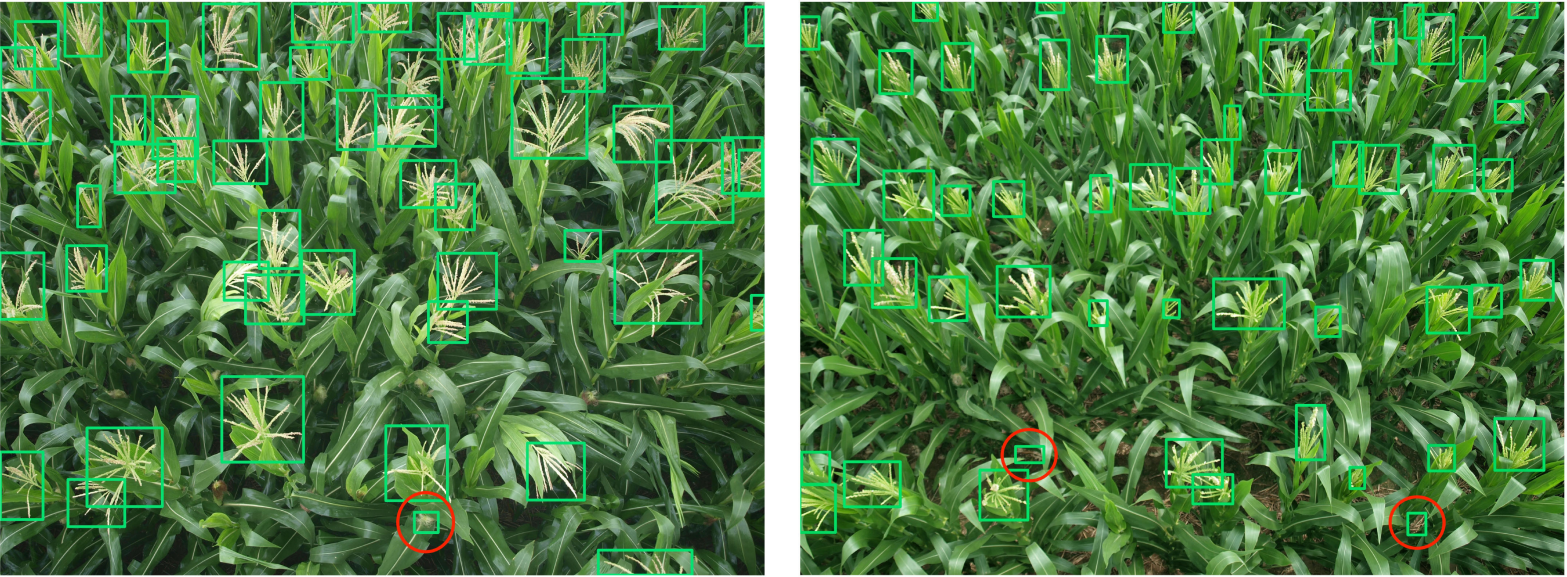
Interference from weeds and maize silks in the accurate detection of maize tassels (red circles indicate false detection).

Computer vision technology has promoted the development of agricultural systems. However, plant counting is not a completely solved problem. We list the advantages and disadvantages of each comparative algorithm as well as their inference time and other parameters in **Table 7**. Detecting objects in natural canopy images with a large number of maize tassels is complex and challenging, as the size, shape, and color of maize tassel at different growth stages vary, and the degree of interference from external information is different. As shown in **Figure 8D**, when the object to be measured is occluded by background information, many underestimations can be observed, which is a popular problem in detection methods that has been continuously improved. Similarly, the scatter plot presented in **Figure 9** illustrates more intuitively that the seemingly good image level mean absolute error (MAE) is usually dominated by underestimations in the detection performance. This is also related to the fact that too little contextual information was considered when constructing the model. The design of lightweight convolutional neural networks aims to achieve faster inference for mobile device applications. However, convolution operations can only capture local information in the window area, which hinders performance improvement in certain scenarios. Introducing self-attention or guided attention into convolution can capture global information well. Guided learning methods can be considered. The principle is to preprocess the incoming image in a way such as training stage to guide the neural network to learn important information more effectively. For example, in maize tassel detection, most of the time, green leaves in the field are the main background information. When weeds inevitably interfere with tassel detection, because the neural network has learned a lot about how to counter information dominated by green leaves and ignored learning about weeds and other interfering information, this will lead to a significant increase in the competitiveness of weeds and filaments in interfering with tassel detection during inference, as shown in **Figure 10**. In the early visual applications in agriculture, Yu et al. (2013) found that the color distribution of monochrome objects changes with brightness on the chromaticity saturation plane, and proposed a crop segmentation method AP-HI, which is insensitive to outdoor brightness and complex environmental elements and can adaptively extract crops. If this adaptive method is applied to suppress some green leaf information in the training images, the learning level of the neural network on green leaves can be reduced, thereby guiding the neural network to learn more about how to counter weeds and other interfering information, enhance the feature competition of the original model, and capture more discriminative feature information. To some extent, this is similar to the visual attention mechanism, which guides the neural network to focus more on certain features by learning the importance of information between features (Hu et al., 2018). Masking is direct method as another option. Pei et al. (2022) used an improved Yolov4 to detect and mask maize rows, and then detected weeds in the masked image. This preprocessing method reduces the imbalance between positive and negative samples and effectively improves detection accuracy.

**Table 7:** Characteristics of different models.

Model	Param (M)	Inference Time (s)	Advantage	Disadvantage
SSD	90.6	0.0152	High real-time performance, easy to train	Many parameters, poor generalization ability
EfficientDet	15.1	0.0417	Fewer parameters, easier to train	Lower real-time performance, lower detection accuracy for dense and small targets
RetinaNet	138.0	0.0401	Has multi-scale detection capability	Lower real-time performance, lower detection accuracy for dense and small targets
TasselNetV3-Seg†	7.5	0.0278	Has global modeling capability	Lower real-time performance
Yolox-nano	3.7	0.0172	High real-time performance, easy to train	Lower multi-scale detection accuracy
CenterNet	124.0	0.0179	Has global modeling capability	Weak domain adaptation ability
Yolov7-tiny	11.7	0.0111	High real-time performance	Weak domain adaptation ability
LFANet-HE	6.0	0.0081	High real-time performance	Domain adaptability is relatively weak
LFANet	6.0	0.0129	Balanced real-time performance and accuracy, strong domain adaptation ability	Parameters need to be improved

In addition, while previous works mainly relied on larger backbone networks or higher-resolution input images to achieve higher accuracy, balancing all dimensions of network width/depth/resolution is crucial when considering both accuracy and efficiency (Tan and Le, 2019; Dollár et al., 2021; Tan and Le, 2021; Wang et al., 2022). Recent studies have shown that carefully designed lightweight networks can achieve comparable performance to their heavy counterparts with much less computational cost (Howard et al., 2017; Tan et al., 2020). Moreover, efficient network design can be further improved by leveraging knowledge distillation, which involves transferring the knowledge learned by a complex model to a simpler one (Hinton et al., 2015; Zagoruyko and Komodakis, 2017; Liu H, et al., 2018; Chen et al., 2020). In agriculture applications, this approach can enable the deployment of more lightweight models on edge devices with limited computational resources, while still maintaining satisfactory accuracy.

Overall, computer vision techniques have greatly facilitated the development of agricultural systems, but challenges remain in accurately counting and detecting objects in complex and cluttered natural scenes. Various strategies such as introducing self-attention or guided attention into convolution operations, incorporating adaptive preprocessing methods, and leveraging efficient network design and knowledge distillation can be explored to improve detection performance and enhance the robustness of agricultural computer vision systems.

## 5 Conclusion

At this stage, designing a lightweight, effective, and easily implementable deep neural network for agricultural application scenarios is both challenging and important. In this study, we propose a novel neural network, TasselLFANet, for accurate and efficient detection and counting of maize tassels in high spatiotemporal image sequences. Our experiments confirm the promising performance of our proposed method compared to existing lightweight models, demonstrating superior performance, flexibility, and adaptability. In practical applications, we offer the following recommendations:

1) For optimal performance, speed, and robustness, we recommend using TasselLFANet.
2) For resource-constrained hardware devices, TasselLFANet-HE is recommended due to its optimal efficiency and reasonable accuracy in most cases.
3) For scenes with strong features during the early stages of plant growth, we recommend using TasselLFANet, which has higher sensitivity to the tasseling stage.
4) In training, enriching the size and environmental diversity of plant imaging can result in a more robust model that adapts to scale and environment transformations.

For future research, we plan to further deploy TasselLFANet in combination with machinery and equipment in agricultural and natural environments, and make targeted improvements based on feedback to meet higher standards and more demanding scenarios. The integration of computer vision technology with agriculture has promoted the advancement of smart agriculture and improved agricultural ecology. We expect that the dataset we release will attract the attention of researchers and facilitate further research on automated monitoring of plant growth. We believe that by collaborating across different fields, we can take the combination of computer vision technology and agriculture to the next level.

## Data availability statement

The original contributions presented in the study are included in the article/**Supplementary Material**. Further inquiries can be directed to the corresponding author.

## Author contributions

ZY and JY contributed to conception and design of the study. CL and XL provided the fields and collected the images. ZY and JY performed the data processing and modelling. JY wrote the first draft of the manuscript. ZY wrote sections of the manuscript. HZ edited the manuscript extensively. ZY and HZ participated in project management and obtained the funding for this study. All authors contributed to the article and approved the submitted version.
