# Supplementary material for: TasselLFANet: a novel lightweight multi-branch feature aggregation neural network for high-throughput image-based maize tassels detection and counting
Source: Front Plant Sci. 2023 Apr 14;14:1158940. doi: 10.3389/fpls.2023.1158940 (PMC10140537; doi:10.3389/fpls.2023.1158940)
Supplement: Supplementary file 1 [file Presentation_1.pdf]

Supplementary Material

TasselLFANet: A Novel Lightweight Multi-Branch Feature Aggregation Neural Network for High-throughput Image-based Maize Tassels Detection and Counting

Zhenghong Yu*, Jianxiong Ye, Cuina Li, Huabing Zhou, Xun Li

*** Correspondence:** Dr. Zhenghong Yu, Email: honger1983@gmail.com

1 Supplementary Video

We provide a demonstration video of the comparative experiment. See website address:

https://youtu.be/NPVdX-MX3es

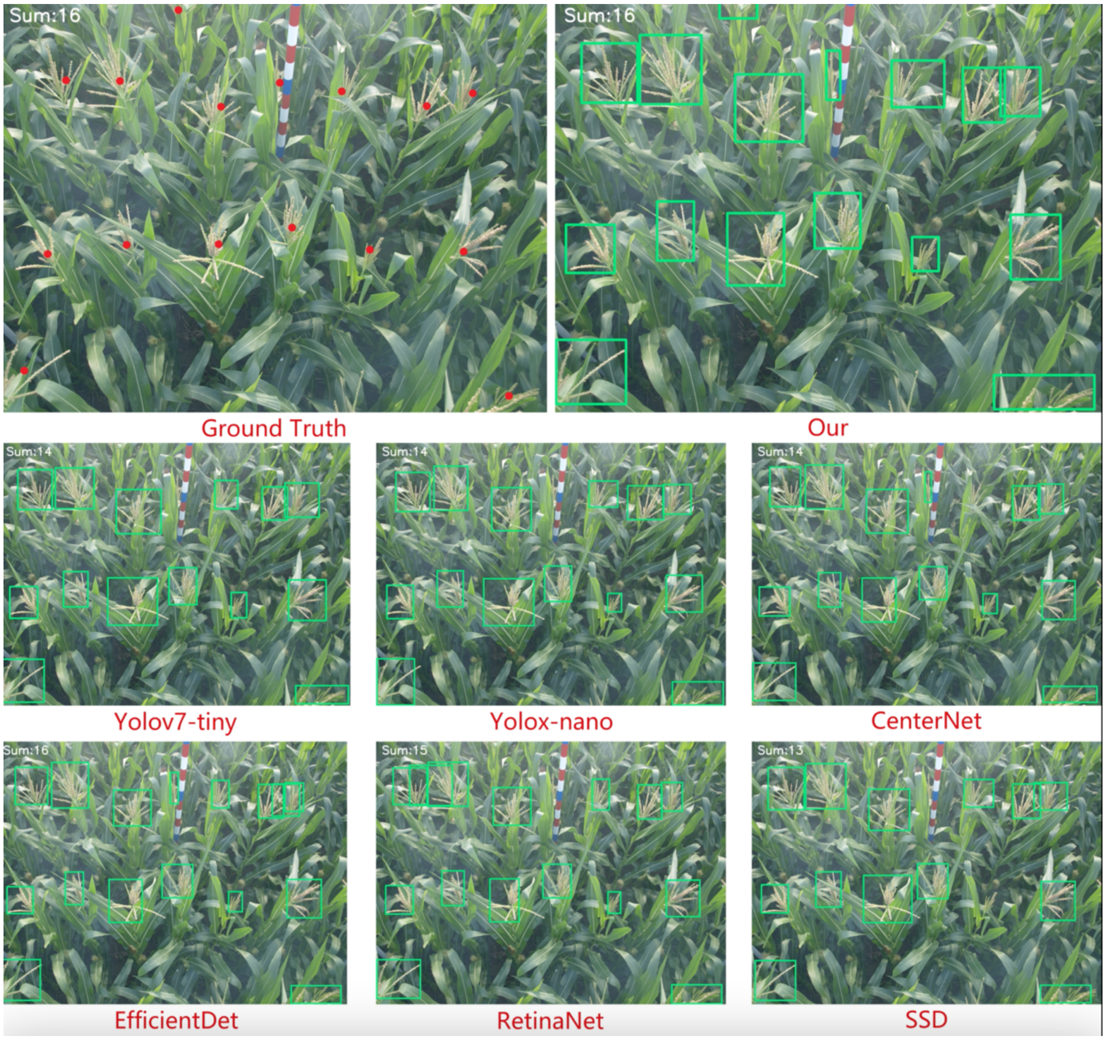

2 Code and Dataset

Please refer to the website for the code of our model:

https://github.com/Ye-Sk/TasselLFANet

The public dataset MrMT we provide can be obtained from the website:

https://github.com/Ye-Sk/MrMT
